# Prognostic implication of IgG4 and IgG1-positive cell infiltration in the lung in patients with idiopathic interstitial pneumonia

**DOI:** 10.1038/s41598-022-13333-8

**Published:** 2022-06-03

**Authors:** Masamichi Komatsu, Hiroshi Yamamoto, Takeshi Uehara, Yukihiro Kobayashi, Hironao Hozumi, Tomoyuki Fujisawa, Atsushi Miyamoto, Tomoo Kishaba, Fumihito Kunishima, Masaki Okamoto, Hideya Kitamura, Tae Iwasawa, Shoichiro Matsushita, Yasuhiro Terasaki, Shinobu Kunugi, Atsuhito Ushiki, Masanori Yasuo, Takafumi Suda, Masayuki Hanaoka

**Affiliations:** 1 First Department of Internal Medicine, Shinshu University School of Medicine, Matsumoto, Japan; 2 Department of Laboratory Medicine, Shinshu University School of Medicine, Matsumoto, Japan; 3 Second Division, Department of Internal Medicine, Hamamatsu University School of Medicine, Hamamatsu, Japan; 4 Department of Respiratory Medicine, Respiratory Center, Toranomon Hospital, Tokyo, Japan; 5 Department of Respiratory Medicine, Okinawa Prefectural Chubu Hospital, Uruma, Japan; 6 Division of Pathology, Okinawa Prefectural Chubu Hospital, Uruma, Japan; 7 Division of Respirology, Neurology, and Rheumatology, Department of Internal Medicine, Kurume University School of Medicine, Kurume, Japan; 8 Division of Respiratory Medicine, Kanagawa Cardiovascular and Respiratory Center, Yokohama, Japan; 9 Department of Radiology, Kanagawa Cardiovascular and Respiratory Center, Yokohama, Japan; 10 Department of Radiology, Yokohama City University Hospital, Yokohama, Japan; 11 Department of Analytic Human Pathology, Nippon Medical School, Tokyo, Japan; 12 Division of Pathology, Nippon Medical School Hospital, Tokyo, Japan; 13 Department of Clinical Laboratory Sciences, Shinshu University School of Health Sciences, Matsumoto, Japan

**Keywords:** Biomarkers, Respiration

## Abstract

Immunoglobulin (Ig) G4-positive cells are rarely observed in the lungs of patients with idiopathic interstitial pneumonias (IIPs). IgG1 may be more pathogenic than IgG4, with IgG4 having both pathogenic and protective roles in IgG4-related disease (IgG4-RD). However, the role of both IgG1 and IgG4 in IIPs remains unclear. We hypothesized that patients with IgG4-positive interstitial pneumonia manifest different clinical characteristics than patients with IgG4-RD. Herein, we identified the correlation of the degree of infiltration of IgG1- and IgG4-positive cells with IIP prognosis, using a Japanese nationwide cloud-based database. We included eighty-eight patients diagnosed with IIPs after multidisciplinary discussion, from April 2009 to March 2014. IgG4-positive cell infiltration was identified in 12/88 patients with IIPs and 8/41 patients with idiopathic pulmonary fibrosis (IPF). Additionally, 31/88 patients with IIPs and 19/41 patients with IPF were diagnosed as having IgG1-positive cell infiltration. IgG4-positive IIPs tended to have a better prognosis. Conversely, overall survival in cases with IgG1-positive IPF was significantly worse. IIPs were prevalent with IgG1- or IgG4-positive cell infiltration. IgG1-positive cell infiltration in IPF significantly correlated with a worse prognosis. Overall, evaluating the degree of IgG1-positive cell infiltration may be prognostically useful in cases of IPF.

## Introduction

Immunoglobulin (Ig) G4-related disease (IgG4-RD) is a systemic disease characterised by an elevated serum IgG4 concentration and IgG4-positive plasma cell infiltration into multiple organs^1,2^. Up to 35% of patients with IgG4-RD present with intrathoracic lesions, including those involving the mediastinal lymph nodes, bronchial walls, and peribronchovascular bundles^3,4^. All lesions in the thorax have been comprehensively classified as IgG4-related respiratory disease (IgG4-RRD)^5^. However, the diagnosis of IgG-RRD is sometimes difficult and requires multidisciplinary discussion (MDD)^6^.

Additionally, various types of idiopathic interstitial pneumonia (IIP) with IgG4-positive plasma cell infiltration in the lungs^7–14^ have been reported. Recently, we reported 16 cases with clinical characteristics of interstitial pneumonia, abundant IgG4-positive cells in the lungs and elevated serum IgG4 levels without extra-thoracic lesions^15^. In most cases, marked numbers of lymphoplasmacytic cells in the fibrous parenchyma were observed. In contrast, none of the 16 patients had either obliterative vasculitis or storiform fibrosis, which are characteristic of IgG4-RD. In addition, progressive fibrosis was observed despite glucocorticoid treatment. We speculate that these cases have IgG4-positive interstitial pneumonias, and should be given an alternative treatment compared to those with IgG4-RRD.

We thought that it was necessary to examine the frequency of IIPs with IgG4-positive cell infiltration in lung tissues and to investigate the clinical significance of IgG4-positive cell infiltration.

Furthermore, whether IgG4 antibodies are active in the pathogenesis of this disease also remains to be elucidated. IgG4 is considered to be a weak-inflammatory or more specifically anti-inflammatory Ig^16^. Recently, it has been suggested that IgG1 has pathogenic roles and IgG4 has both pathogenic and protective roles in IgG4-related pancreatitis (autoimmune pancreatitis)^17^. We speculated that IIPs with abundant IgG1-positive cells may have a poor prognosis regardless of the degree of IgG4-positive cells. However, the roles of IgG1 and IgG4 in patients with IIPs remain unclear. Therefore, it was necessary to examine the clinical characteristics of patients with IIPs with or without IgG1 as well as IgG4-positive cell infiltration.

In this study, we retrospectively analysed the presence of IgG-, IgG1-, and IgG4-positive cells by immunostaining surgical lung biopsy (SLB) specimens obtained from patients with IIPs. The patients’ diagnoses were confirmed by MDD. The current study is the largest survey of IgG4-positive cells in SLB specimens for IIPs and the first one evaluating IgG1-positive cell infiltration in SLB specimens for IIPs.

## Results

### Patient characteristics and multidisciplinary discussion

The MDD diagnoses of the 88 patients are shown in Table 1. The most prevalent MDD diagnosis was IPF (41 patients), followed by unclassifiable IIPs (28 patients). The patient characteristics for IIPs and IPF are described in Table 2. The median ages of diagnosis of IIPs and IPF were 65.0 and 66.0 years, respectively. In patients with IIP, serum IgG4 was analysed in 24 patients, and the median level of serum IgG4 was 40.5 mg/dL.

**Table 1:** IgG1- and IgG4-positive cells in the lungs with IIPs.

MDD diagnosis	IPF	iNSIP	COP	DIP	PPFE	Unclassifiable IIPs
	41	9	1	6	3	28
IgG1 score 0; < 5 (per HPF)	7	3	1	1	2	12
IgG1 score 1; 5 to 10 (per HPF)	6	1	0	0	1	3
IgG1 score 2; 11 to 30 (per HPF)	9	2	0	3	0	6
IgG1 score 3; > 30 (per HPF)	19	3	0	2	0	7
IgG4 score 0; < 5 (per HPF)	29	8	1	4	3	22
IgG4 score 1; 5 to 10 (per HPF)	4	0	0	1	0	4
IgG4 score 2; 11 to 30 (per HPF)	5	1	0	1	0	2
IgG4 score 3; > 30 (per HPF)	3	0	0	0	0	0

Data are presented as N.

We defined interstitial pneumonia when IgG1-positive cell infiltration was > 30/HPF and IgG4-positive cell infiltration was > 10/HPF.

*COP* cryptogenic organising pneumonia, *DIP* desquamative interstitial pneumonia, *HPF* high-power field, *Ig* immunoglobulin, *IIP* idiopathic interstitial pneumonia, *iNSIP* idiopathic non-specific interstitial pneumonia, *IPF* idiopathic pulmonary fibrosis, *MDD* multidisciplinary discussion, *PPFE* pleuroparenchymal fibroelastosis.

**Table 2:** Characteristics of patients with IIPs and IPF.

	IIPs (N = 88)	IPF (N = 41)
Age, years	65.0 (20.0–78.0)	66.0 (43.0–78.0)
Sex, male/female	54 (60.8%)/34 (39.2%)	24 (58.5%)/17 (41.5%)
Smoking, y/n	48 (54.5%)/40 (45.5%)	21 (1.2%)/20 (48.8%)
**MDD**		
IPAF/non-IPAF	28/69	8/33
**Immunohistochemistry**		
IgG-positive cell/HPF	22 (0–212)	28 (3–153)
IgG1-positive cell/HPF	14 (0–173)	30 (0–115)
IgG4-positive cell/HPF	2 (0–75)	1 (0–75)
IgG1-positive cell/IgG-positive cell	67.3 (0.0–350)	73.9 (0.0–204.5)
IgG4-positive cell/IgG-positive cell	6.5 (0.0–130.0)	5.8 (0.0–64.1)
**Laboratory**		
LDH, U/L	231 (132–447)	239 (185–2341)
KL-6, U/mL	1257 (151–16,120)	1051 (338–4350)
SP-D, ng/mL	220 (45–1350)	202 (55–401)
serum IgG4, mg/dL	40.5 (8.0–372.0) N = 24	59.6 (13.0–372.0) N = 9
**Pulmonary function**		
FVC, L	2.46 (0.71–4.62)	2.34 (0.71–3.76)
%FVC, %predicted	78.0 (30.6–126.5)	77.9 (30.6–126.5)
%DLco, %predicted	65.0 (25.7–154.0)	60.7 (27.7–114.8)
**6-min walk test**		
Distance, meter	475 (200–660)	450 (200–650)
min SpO2, %	93 (79–96)	93 (79–96)
**Radiographic pattern**		
Definite UIP/Possible UIP/Inconsistent with UIP	3 (3.4%)/43 (48.9%)/40 (45.5%)	3 (7.3%)/33 (80.5%)/ 5 (12.2%)
**Pathological pattern**		
Definite UIP/Probable UIP/Possible UIP/Not UIP	12 (13.6%)/35 (39.8%)/18 (20.5%)/17 (19.3%)	10 (24.4%)/20 (48.8%)/5 (12.2%)/0 (0.0%)
**Treatment**		
Corticosteroids	51 (58.0%)	22 (53.7%)
Immunosuppressants	23 (26.1%)	8 (19.5%)
Antifibrotic drugs	26 (29.5%)	19 (46.3%)
Follow-up, years	3.93 (0.05–12.59)	3.30 (0.15–12.43)
**Clinical event**		
Acute exacerbation	22 (25.0%)	11 (26.8%)
Lung cancer	3 (3.4%)	2 (4.9%)
Dead cases	27 (30.7%)	16 (39.0%)
**Cause of death**		
Chronic respiratory failure	13	7
Acute exacerbation	6	4
Infection	4	2
Others	4	3

Data are presented as the median (range) or N (%).

*DLco* diffusing capacity of the lung for carbon monoxide, *FVC* forced vital capacity, *HPF* high-power field, *Ig* immunoglobulin, *IIP* idiopathic interstitial pneumonia, *IPAF* interstitial pneumonia with autoimmune features, *IPF* idiopathic pulmonary fibrosis, *KL-6* Krebs von Lungen-6, *LDH* lactate dehydrogenase, *MDD* multidisciplinary discussion, *SP* surfactant protein, *UIP* usual interstitial pneumonia, *y/n* yes/no.

The median follow-up period in cases of IIPs was 3.93 years (range 0.05–12.59). During the follow-up period, acute exacerbation and lung cancer developed in 22 patients (25.0%) and three patients (3.4%), respectively. None of the patients developed extra-thoracic IgG4-RD. Thirteen patients died of chronic respiratory failure, six patients died from an acute exacerbation, and four patients died from an infection in the follow-up period. In IIPs, the median numbers of positive cells per high-power field (HPF) in the immunohistochemistry for IgG, IgG1, and IgG4 were 22, 14, and 2, respectively. The IgG1 + /IgG + ratio and IgG4 + /IgG + ratio were 67.3, and 6.5, respectively.

In the IPF group, the median numbers of positive cells per HPF in the immunohistochemistry analysis for IgG, IgG1, and IgG4 were 28, 30, and 1, respectively. The IgG1 + /IgG + ratio and IgG4 + /IgG + ratio were 73.9 and 5.8, respectively.

The degrees of IgG1-positive cells and IgG4-positive cells in cases of IIPs are summarised in Table 1. Furthermore, there was a positive correlation between serum IgG4 and IgG4-positive cells/HPF (rs = 0.693, *p* < 0.01), while there was a weak positive correlation between IgG1-positive cells and IgG4-positive cells (rs = 0.477, *p* = 0.02) (Supplementary Figs. S1, S2 online).

### Definition of IgG1- or IgG4-positive IIPs

In this study, if the IgG4-positive cells were scored 2 or more (> 10/HPF), IgG4-positive IIP was diagnosed with reference to the proposed IgG4-RRD diagnostic criteria^5^. Among patients with IIP, 12 of 88 patients (13.6%) were diagnosed as having IgG4-positive IIPs; among patients with IPF, 8 of 41 patients (19.5%) were diagnosed as having IgG4-positive IPF, according to this definition. Similarly, one of nine idiopathic non-specific interstitial pneumonia (iNSIP) patients (11.1%), and one of six desquamative interstitial pneumonia (DIP) patients (16.7%), were diagnosed as having IgG4-positive IIPs (Table 1).

In this study, the cells with a score greater than 3 (> 30/HPF) with reference to the median number of IgG1-positive cells in IPF were defined as IgG1-positive cells. Among the 88 patients with IIP, 31 (35.2%) were diagnosed as having IgG1-positive IIPs; among the 41 patients with IPF, 19 (46.3%) were diagnosed as having IgG1-positive IPF. Similarly, three of nine (33.3%) patients with iNSIP, and two of six (33.3%) patients with DIP were diagnosed as having IgG1-positive IIPs (Table 1).

### Comparison of IIPs and IPF with or without IgG4-positive cells

We compared the patients with IIPs and IPF with or without IgG4-positive cell infiltration (IgG4-positive or negative IIPs, as well as IgG4-positive or negative IPF).

The characteristics of the groups are summarised in Table 3. In both the IIPs and IPF groups, the serum levels of IgG4 were significantly higher in IgG4-positive patients than in IgG4-negative patients. In immunohistochemistry, IgG- and IgG1-positive cells/HPF, and IgG4 + /IgG + ratio were significantly high in both IgG4-positive groups. In the physiological tests, the predicted percentage of the diffusing capacity of the lung for carbon monoxide (DLco) was significantly lower in IgG4-positive IPF than in IgG4-negative IPF. The frequency of patients who fulfilled the criteria for diagnosis of interstitial pneumonia with autoimmune features (IPAF) did not differ between the two groups. Detailed clinical features and serum autoantibody positivity are listed in Supplementary Table S1 online.

**Table 3:** Characteristics of patients with IIPs and IPF with or without IgG4-positive cell infiltration.

	IgG4-positive IIPs (N = 12)	IgG4-negative IIPs (N = 76)	*p-*value	IgG4-positive IPF (N = 8)	IgG4-negative IPF (N = 33)	*p-*value
Age, years	68.0 (20.0–78.0)	64.5 (25.0–78.0)	0.627	68.0 (61.0–74.0)	66.0 (43.0–78.0)	0.502
Sex, male/female	6 (50.0%)/6(50.0%)	48 (63.2%)/28(36.8%)	0.525	3 (37.5%)/5 (62.5%)	21 (63.6%)/12 (36.4%)	0.241
Smoking, y/n	5 (41.7%)/7(58.3%)	43 (56.6%)/33 (43.4%)	0.335	3 (37.5%)/5 (62.5%)	18 (54.5%)/15 (45.5%)	0.454
MDD						
IPAF/non-IPAF	6/6	19/57	0.092	3/5	5/28	0.172
**Immunohistochemistry**						
IgG-positive cell/HPF	75 (10–153)	19 (0–212)	**0.002**	87 (44–153)	21 (3–151)	** < 0.001**
IgG1-positive cell/HPF	43 (3–115)	12 (0–173)	**0.001**	60 (11–115)	15 (0–97)	** < 0.001**
IgG4-positive cell/HPF	21 (11–75)	1 (0–10)	** < 0.001**	29 (11–75)	1 (0–10)	** < 0.001**
IgG1-positive cell/IgG-positive cell	84.1 (9.4–350.0)	62.5 (0.0–187.5)	**0.015**	71.0 (9.4–204.5)	77.8 (0.0–187.5)	0.763
IgG4-positive cell/IgG-positive cell	39.1 (17.2–130.0)	4.7 (0.0–71.4)	** < 0.001**	35.4 (17.2–64.1)	3.6 (0.0–33.3)	** < 0.001**
**Laboratory**						
LDH, U/L	252 (162–295)	223 (132–447)	0.324	250 (231–289)	230 (182–447)	0.366
KL-6, U/mL	1923 (644–16,120)	1110 (151–8137)	**0.009**	1923 (644–16,120)	907 (151–8137)	**0.024**
SP-D, ng/mL	376 (66–1350)	216 (45–786)	**0.004**	489 (17–1350)	220 (69–533)	** < 0.001**
Serum IgG4, mg/dL	228.5 (85.0–372.0) N = 2	35.6 (8.0–177.0) N = 22	** < 0.001**	228.5 (85.0–372.0) N = 2	40.6 (13.0–124.0) N = 7	**0.038**
**Pulmonary function**						
FVC, L	2.38 (1.37–3.80)	2.46 (0.71–4.62)	0.623	1.95 (1.37–3.26)	2.43 (0.71–3.76)	0.466
%FVC, %predicted	75.9 (58.8–119.0)	78.8 (30.6–126.5)	0.875	75.9 (58.8–103.5)	79.7 (30.6–126.5)	0.889
%DLco, %predicted	56.8 (27.7–83.1)	67.0 (25.7–154.0)	0.076	48.8 (27.7–61.4)	67.4 (36.2–114.8)	**0.009**
**6-min walk test**						
Distance, meter	447 (230–650)	484 (200–660)	0.480	440 (230–650)	460 (200–640)	0.577
min SpO2, %	93 (79–94)	92 (79–96)	0.745	93 (79–94)	93 (79–96)	0.371
GAP index						0.047
Stage I	N.E	N.E		3 (42.9%)	21 (84.0%)	
Stage II	N.E	N.E		4 (57.1%)	4 (16.0%)	

Data are presented as the median (range), or N (%). Bold font: *p*-value < 0.05.

*DLco* diffusing capacity of the lung for carbon monoxide, *FVC* forced vital capacity, *HPF* high-power field, *Ig* immunoglobulin, *IIP* idiopathic interstitial pneumonia, *IPAF* interstitial pneumonia with autoimmune features, *IPF* idiopathic pulmonary fibrosis, *KL-6* Krebs von Lungen-6, *LDH* lactate dehydrogenase, *MDD* multidisciplinary discussion, *SP* surfactant protein, *y/n* yes/no.

**Figure 1:**
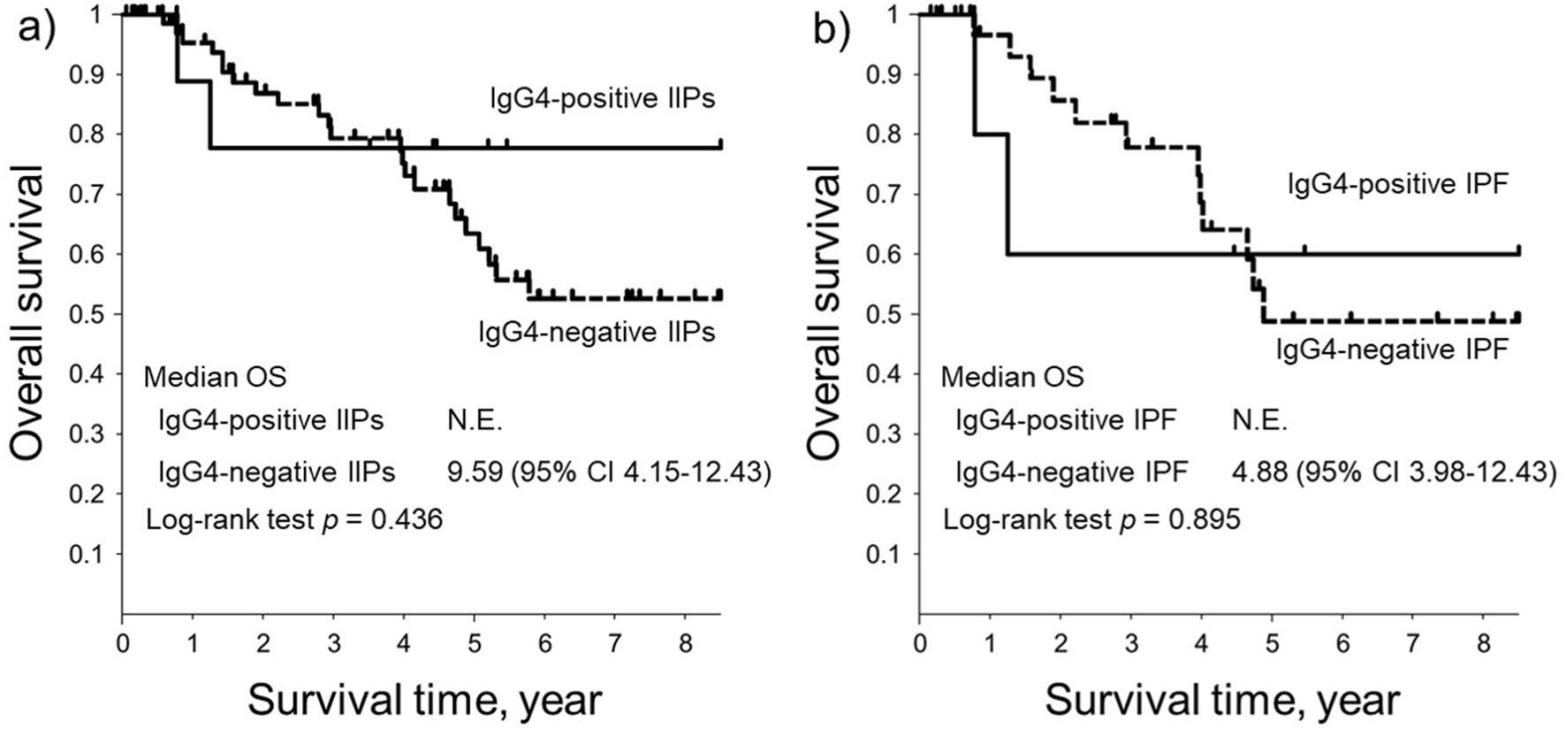
Survival curves of patients with either IIPs or IPF with or without IgG4-positive cell infiltration. (**a**) The Kaplan–Meier curve of the overall survival of patients with idiopathic interstitial pneumonias with or without IgG4-positive cell infiltration in the lungs (IgG4-positive or negative IIPs). (Solid line: IgG4-positive IIPs, Broken line: IgG4-negative IIPs). (**b**) The Kaplan–Meier curve of the overall survival of patients with idiopathic pulmonary fibrosis with or without IgG4-positive cell infiltration in the lungs (IgG4-positive or negative IPF). (Solid line: IgG4-positive IPF, Broken line: IgG4-negative IPF). *CI* confidence interval, *Ig* immunoglobulin, *IIP* idiopathic interstitial pneumonia, *IPF* idiopathic pulmonary fibrosis, *N.E.* not evaluable, *OS* overall survival.

The Kaplan–Meier curves of overall survival (OS) for patients with or without IgG4-positive cells are shown in Fig. 1. Although there were no statistical differences between the two groups, the prognosis of IgG4-positive IIPs tended to be better than that of IgG4-negative IIPs (Fig. 1a). In contrast, there were no differences in survival for patients with IPF between the groups of patients with or without IgG4-positive cells (Fig. 1b).

### Comparison of IIPs and IPF with or without IgG1-positive cells

We compared the patients with IIPs and IPF with or without IgG1-positive cell infiltration (IgG1-positive, or negative IIPs, as well as IgG1-positive or negative IPF). The characteristics of the groups are summarised in Table 4. In immunohistochemistry, IgG- and IgG1-positive cells/HPF, and IgG1 + /IgG + ratio were significantly high in both IgG1-positive groups. There were no differences between the two groups in the baseline values obtained from the pulmonary function tests. In the 6-min walk test, there was no difference in the distance, but the lowest arterial oxygen saturation measured with pulse oximetry was significantly lower in the IgG1-positive IPF group than in the IgG1-negative IPF group.

**Table 4:** Characteristics of patients with IIPs and IPF with or without IgG1-positive cell infiltration.

	IgG1-positive IIPs (N = 31)	IgG1-negative IIPs (N = 57)	*p-*value	IgG1-positive IPF (N = 19)	IgG1-negative IPF (N = 22)	*p-*value
Age, years	64.0 (20.0–75.0)	66.0 (25.0–78.0)	0.185	66.0 (54.0–74.0)	67.5 (43.0–78.0)	0.838
Sex, male/female	18 (58.1%)/13 (41.9%)	36 (63.2%)/21 (36.8%)	0.639	10 (52.6%)/9 (47.4%)	14 (63.6%)/8 (36.4%)	0.476
Smoking, y/n	6 (42.9%)/16 (51.6%)	33 (57.9%)/24 (42.1%)	0.392	9 (47.4%)/10 (52.6%)	12 (54.5%)/10 (45.5%)	0.647
**MDD**						
IPAF/non-IPAF	10/21	15/42	0.555	5/14	3/19	0.4361
**Immunohistochemistry**						
IgG-positive cell/HPF	74 (10–212)	14 (0–117)	** < 0.001**	67 (20–153)	17 (3–117)	** < 0.001**
IgG1-positive cell/HPF	49 (32–173)	6 (0–30)	** < 0.001**	71 (32–115)	9 (0–30)	** < 0.001**
IgG4-positive cell/HPF	5 (0–65)	1 (0–30)	**0.004**	6 (0–65)	1 (0–75)	0.102
IgG1-positive cell/IgG-positive cell	94.7 (36.2–350.0)	42.3 (0.0–187.5)	** < 0.001**	94.7 (36.2–204.5)	51.1 (0.0–187.5)	** < 0.001**
IgG4-positive cell/IgG-positive cell	10.0 (0.0–130.0)	5.3 (0.0–85.7)	0.709	11.7 (0.0–42.5)	0.7 (0.0–64.1)	0.131
**Laboratory**						
LDH, U/L	247 (162–361)	223 (132–447)	0.127	247 (193–355)	224 (182–447)	0.380
KL-6, U/mL	1508 (151–16,120)	1175 (221–5646)	0.101	1508 (151–16,120)	976 (348–3030)	0.092
SP-D, ng/mL	293 (74–1350)	195 (45–786)	**0.023**	365 (74–1350)	155 (69–533)	**0.018**
serum IgG4, mg/dL	49.0 (8.0–372.0) N = 4	40.5 (9.3–177.0) N = 20	0.122	85.0 (13.0–372.0) N = 3	50.1 (16.3–124.0) N = 6	0.246
**Pulmonary function**						
FVC, L	2.45 (0.71–3.64)	2.46 (1.06–4.62)	0.317	2.28 (0.71–3.31)	2.39 (1.06–3.76)	0.475
%FVC, %predicted	77.4 (30.6–110.4)	79.0 (31.5–126.5)	0.223	74.6 (30.6–110.4)	81.8 (31.5–126.5)	0.506
%DLco, %predicted	59.6 (25.7–124.5)	67.0 (27.7–154.0)	0.598	57.8 (36.2–114.8)	66.7 (27.7–108.0)	0.633
**6-min walk test**						
Distance, m	469 (230–650)	500 (200–660)	0.566	455 (230–650)	450 (200–640)	0.731
min SpO2, %	92 (79–95)	93 (83–96)	0.223	90 (79–95)	93 (88–96)	**0.013**
GAP index						0.703
Stage I	N.E	N.E		10 (71.4%)	14 (77.8%)	
Stage II	N.E	N.E		4 (28.6%)	4 (22.2%)	

Data are presented as the median (range) or N (%). Bold font: *p*-value < 0.05.

*DLco* diffusing capacity of the lung for carbon monoxide, *FVC* forced vital capacity, *GAP* gender, age, physiological variable, *HPF* high-power field, *Ig* immunoglobulin, *IIP* idiopathic interstitial pneumonia, *IPAF* interstitial pneumonia with autoimmune features, *IPF* idiopathic pulmonary fibrosis, *KL-6* Krebs von Lungen-6, *LDH* lactate dehydrogenase, *MDD* multidisciplinary discussion, *N.E.* not evaluable; *SP* surfactant protein, *y/n* yes/no.

**Figure 2:**
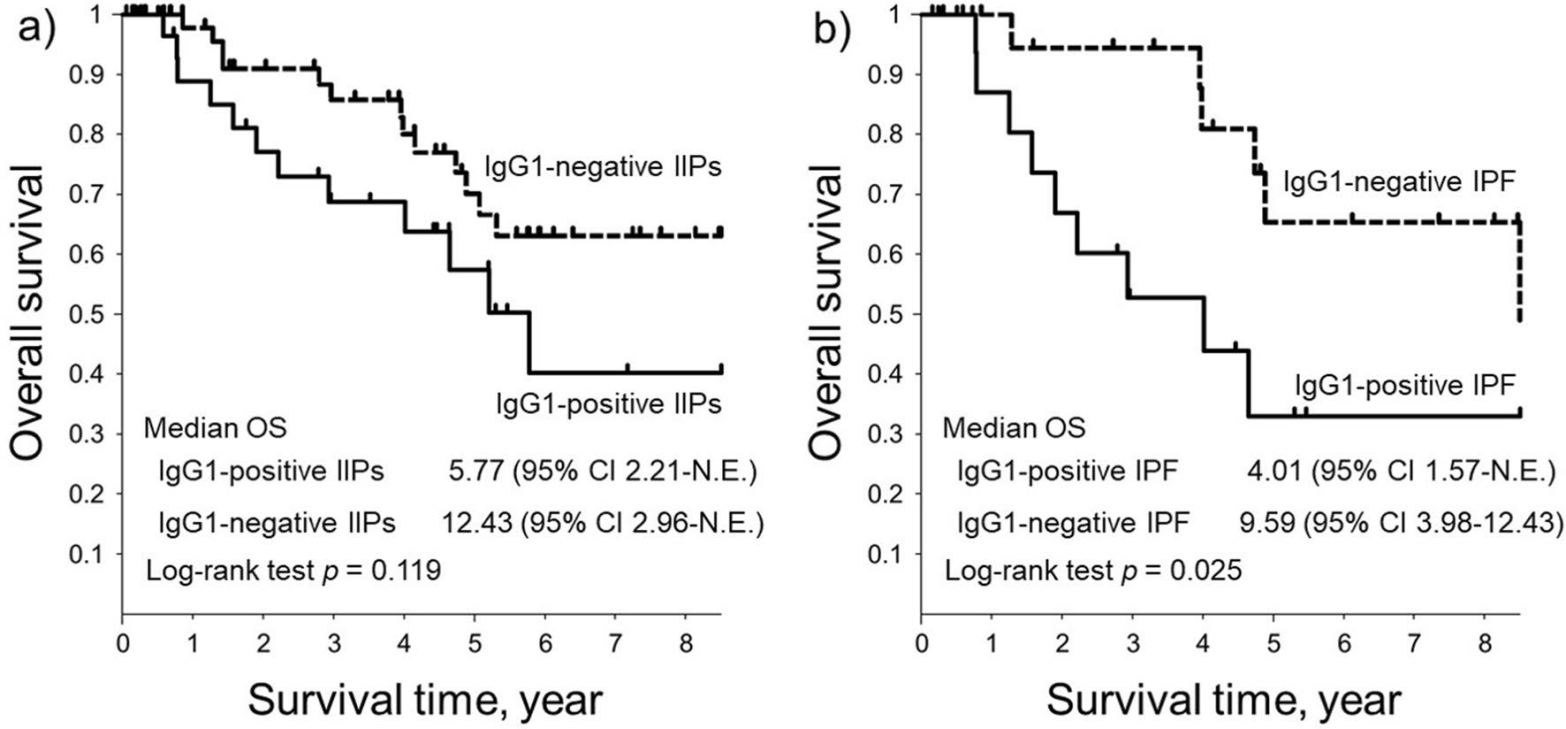
Survival curves of patients with either IIPs or IPF with or without IgG1-positive cell infiltration. (**a**) The Kaplan–Meier curve of the overall survival of patients with idiopathic interstitial pneumonias with or without IgG1-positive cell infiltration in the lungs (IgG1-positive or negative IIPs). (Solid line: IgG1-positive IIPs, Broken line: IgG1-negative IIPs). (**b**) The Kaplan–Meier curve of the overall survival of patients with idiopathic pulmonary fibrosis with or without IgG1-positive cell infiltration in the lungs (IgG1-positive or negative IPF). (Solid line: IgG1-positive IPF, Broken line: IgG1-negative IPF). *CI* confidence interval, *Ig* immunoglobulin, *IIP* idiopathic interstitial pneumonia, *IPF* idiopathic pulmonary fibrosis, *N.E.* not evaluable, *OS* overall survival.

The Kaplan–Meier curves of OS for the IgG1-positive or IgG1-negative group are shown in Fig. 2. There were statistically significant differences in survival between IgG1-positive IPF and IgG1-negative IPF (Fig. 2b).

The univariate Cox proportional hazards regression analysis showed that IgG1-positive cell > 30/HPF (hazard ratio [HR] 3.411, 95% confidence intervals [CI] 1.127–10.322; *p* = 0.030) and the incidence of acute exacerbation (HR 3.241, 95% CI 1.123–9.356; *p* = 0.030) were associated with a high risk of death. The multivariate analysis showed that IgG1-positive cell > 30/HPF (HR 4.069, 95% CI 1.252–13.230; *p* = 0.020) and the incidence of acute exacerbation (HR 4.073, 95% CI 1.370–12.114; *p* = 0.012) were independently associated with a high risk of death (Table 5).

**Table 5:** Univariate and multivariate Cox proportional hazard regression analysis of the death risk in patients with IPF.

Variable	Univariate			Multivariate		
	HR	95% CI	*p-*value	HR	95% CI	*p-*value
Age, years	1.684	0.779–3.638	0.185			
Sex	0.691	0.242–1.971	0.489			
IgG1-positive cell > 30/HPF or < = 30/HPF	3.411	1.127–10.322	**0.030**	4.069	1.252–13.230	**0.020**
IgG4-positive cell > 10 HPF or < = 10/HPF	0.904	0.203–4.035	0.895			
KL-6, U/mL	1.000	1.000–1.000	0.481			
SP-D, ng/mL	1.001	0.999–1.002	0.568			
%FVC	1.011	0.504–2.028	0.974			
%DLco	1.071	0.396–2.898	0.892			
GAP stage	0.962	0.230–4.034	0.958			
Usage of antifibrotic agents	0.131	0.738–10.331	0.131			
Acute exacerbation	3.241	1.123–9.356	**0.030**	4.073	1.370–12.114	**0.012**

Bold font: *p*-value < 0.05.

*CI* confidence interval, *DLco* diffusing capacity of the lung for carbon monoxide, *FVC* forced vital capacity, *GAP* gender, age, physiological variable, *HPF* high-power field, *HR* hazard ratio, *Ig* immunoglobulin, *IPF* idiopathic pulmonary fibrosis, *KL-6* Krebs von Lungen-6, *SP* surfactant protein.

## Discussion

To the best of our knowledge, this is the most comprehensive report on the prevalence of IgG4-positive cell infiltration in SLB specimens of patients with IIPs and the first study of IgG1-positive cell infiltration in these specimens. We found that 19.5% of IPF, 11.1% of iNSIP, and 16.7% of patients with DIP had IgG4-positive IIPs. We also found that patients with IgG4-positive IIPs had a tendency toward a better survival rate than the IgG4-negative IIPs. In addition, patients with IgG1-positive IPF had a significantly worse survival rate than patients with IgG1-negative IPF. This trend was more pronounced when the threshold was changed to 40 cells/HPF (Supplementary Fig. S3 online). Thus, evaluation of IgG1- and IgG4-positive cell infiltration in IIPs might be a useful prognostic tool.

Shrestha et al. reported that IgG4-positive cells > 10/HPF were found in 25% of usual interstitial pneumonia (UIP) and 10% of NSIP^18^. This frequency of IgG4-positive plasma cells in IIPs is comparable to the findings in our study. Ikeda et al. reported that IIPs with abundant IgG4-positive cells had a good response to corticosteroid monotherapy^19^. In the present study, the prognosis of IgG4-positive IIPs tended to be better than that of IgG4-negative IIPs. Further investigation remains warranted to clarify the prognosis of IIPs with or without IgG4-positive cell infiltration.

Recently, we defined IgG4-positive interstitial pneumonia as a condition with elevated serum IgG4 concentrations (≥ 135 mg/dL) and infiltration of abundant IgG4-positive plasma cells (IgG4 + /IgG + ratio > 40%; IgG4-positive cells > 10 per HPF)^15^. Herein, 6 of 88 patients with IIP and 3 of 41 patients with IPF fulfilled both conditions of > 40% IgG4 + /IgG + ratio and > 10 IgG4-positive cells per HPF. Notably, none of the patients developed IgG4-RD. It was plausible that IgG4-positive cells were expressed in IIPs independent of IgG4-RD, although only a few patients were evaluated for serum IgG4 concentration.

On the contrary, IgG1-positive IPF showed a significantly worse survival rate than did IgG1-negative IPF. In immunohistochemistry, IgG1-positive cells are IgG1-positive plasma cells or lymphoid cells containing IgG1. It might be considered that IgG1-positive IPF means IPF involving B cells. Although a precise understanding of the relationship between B cells and IPF remains elusive, there are some existing reports on B cells and IPF^20^. Xue et al. reported that patients with IPF who had enriched B lymphocyte stimulating factor (BLyS) had a worse survival rate than those with lower levels of BLyS^21^. Although further research is warranted, IgG1-positive cells in SLB specimens may be a prognostic indicator of patients with IPF. Therefore, the use of antifibrotic agents in the early stage of IPF or new therapeutic strategies may be required in cases of IPF that also have abundant IgG1-positive cells in the lung. However, further studies are needed to clarify this problem.

In this study, the evaluation of IgG1-positive cells was performed at the sites with abundant IgG4-positive cells. There was no consistent trend in the degree of infiltration by IgG1 and IgG4-positive cells. The present study did not prove that IgG1 is responsive to IgG4. In the future, it will be necessary to evaluate whether IgG4 is found around fibrotic foci or fibrosis to assess whether IgG4 plays a protective role. On the other hand, it has been suggested that abundant IgG1-positive cells, regardless of IgG4-positive cells, is considered a poor prognostic factor of IPF.

The pathogenicity of IgG4-RD remains to be elucidated. It also remains to be determined whether IgG4 antibodies are active in the pathogenesis of IgG4-RD. IgG4 cannot activate the classical complement pathway^22^ and has a low affinity for Fc receptors^23^. IgG4 is considered weak-inflammatory; rather it has the potential to cause a reduction in the immune activation of Igs^16^. Shiokawa et al. revealed that subcutaneous injections of IgG from patients with autoimmune pancreatitis (pancreatic lesion of IgG4-RD) led to organ injuries in a mouse model^17^. Organ injury was also induced by injecting IgG1 and IgG4 from these patients into mice; IgG1 injection caused more destructive changes compared to IgG4. Moreover, organ destruction was more effectively inhibited by simultaneous injections of patient-derived IgG1 and IgG4 than by injection of patient-derived IgG1 alone. These observations suggest that IgG4 antibodies act against pathogenic IgG1 antibodies. Fujimoto et al. reported that in patients with stage I squamous cell lung cancers, IgG4-positive cell infiltration around the tumour in the resected lung specimens corresponded with a favourable prognosis^24^. These phenomena are compatible with our findings that IIPs with IgG4-positive cell infiltration showed a tendency toward a better prognosis and that IPF with IgG1-positive cell infiltration had a significantly worse prognosis. IgG1 and IgG4 immunostaining may be beneficial for IIPs because it may predict prognosis and inform therapeutic management decisions.

This study has several limitations. The nationwide, cloud-based database does not include serum IgG or IgG4 levels; therefore, we obtained these from the clinical records of each institution. Almost all institutions in this study did not routinely test for serum IgG and IgG4; thus, we could only obtain a small sample number. Additionally, the study was not large enough to reach definite conclusions on the prognosis of IIPs with or without IgG4-positive cell infiltration. Despite these limitations, to the best of our knowledge, this is the first report describing IgG-, IgG1-, and IgG4-positive cell infiltration in SLB specimens for IIPs diagnosed by a centralised MDD.

In summary, we revealed the prevalence of IgG4-positive cell infiltration in SLB specimens from patients with IIPs in Japan. The prognosis of IIPs with IgG4-positive cell infiltration tended to be better than those without IgG4-positive cell infiltration. Furthermore, IPFs with IgG1-positive cell infiltration had a significantly worse prognosis than those without IgG1-positive cell infiltration. Overall, evaluations of the degree of IgG1-positive cell infiltration may be prognostically useful in cases of IPF.

## Methods

### Study subjects

This study is a secondary survey of data from a retrospective study^25^. We used a nationwide cloud-based integrated database of IIPs after centralised MDD. Details of the database have been described previously^25^. Information on 524 patients who underwent SLB from April 2009 to March 2014 was collected from 39 institutions throughout Japan. The cloud-based database was constructed including clinico-radiolo-pathological data. Web-based MDD was performed using the database.

In this study, we enrolled 88 patients from five institutions for whom re-pathological analysis was possible. We reviewed the glass slides of SLB from the 88 cases. The institutional review board approved the study protocol (the Ethics Committee of the Shinshu University School of Medicine, Approval Number: 4205) and each institution approved the study protocol (the Ethics Committee of the Hamamatsu University School of Medicine, Approval Number: 2019-0009; Toranomon Hospital, Approval Number: 1773; Prefectural Chubu Hospital, Approval Number: 2018-120; Kurume University School of Medicine, Approval Number: 18221). This study was performed in accordance with the Declaration of Helsinki and its subsequent amendments. The need for obtaining patient written informed consent was waived, owing to the retrospective nature of this study.

### Clinical, laboratory, and physiology findings

Clinical information was obtained from the database for each patient. Although serum levels of IgG, IgG1, and IgG4 were not included in the database, the serum level of IgG4 for 24 of the 88 patients was obtained from the clinical records from each institution. The incidence of acute exacerbation and lung cancer was also obtained from the database. Inadequate items in the database were excluded.

In our cohort, non-IIPs such as sarcoidosis, hypersensitivity pneumonia, or collagen vascular disease-associated interstitial pneumonia were excluded by MDD. Clinical, serologic, and morphological domains were evaluated based on the criterion of IPAF^26^.

Sex, age, physiological (e.g., forced vital capacity [FVC] and DLco) variables, and GAP index were calculated in the IPF group^27^.

Clinical characteristics of IgG4-positive or negative IIPs, as well as IgG1-positive or negative IIPs, were analysed. In addition, we analysed clinical characteristics of IgG4-positive or negative IPF, as well as IgG1-positive or negative IPF.

### Imaging

All high-resolution computed tomography (HRCT) chest images obtained within three months of the SLB were analysed. The radiologists determined the HRCT pattern of IIPs according to the 2011 guidelines^28^ of IPF: definite UIP, possible UIP, or inconsistent with UIP pattern.

### Histopathological findings

The pathologists classified the samples from the SLB according to the 2011 guidelines of IPF classification^28^: definite UIP, probable UIP, possible UIP, and not UIP pattern. The pathologists also determined the histological patterns of IIPs according to the statement of classification for IIPs^29^.

In this study, SLB specimens were stained with haematoxylin–eosin and immuno-stained for IgG, IgG1, and IgG4. IgG immunohistochemical staining was performed using monoclonal IgG antibody (Leica, Lincolnshire, IL, USA). IgG1 and IgG4 immunohistochemical staining were performed using polyclonal antibodies against IgG1 and IgG4 (Binding Site, Birmingham, UK). The areas with the highest density of IgG4-positive cells, identified using immunostaining, were observed under a low-power field. A trained pathologist (T.U.) counted the IgG4-positive cells per HPF. IgG-positive cells/HPF and IgG1-positive cells/HPF were also counted in the same areas where IgG4-positive cells were evaluated. Representative pathological findings are shown in Supplementary Fig. S4 online. The ratio of IgG1-positive cells to IgG-positive cells (IgG1 + /IgG +) as well as IgG4 + /IgG + was also evaluated. IgG4-positive cells were scored according to the following criteria: IgG4 score 0, < 5/HPF; score 1, 5 to 10/HPF; score 2, 11 to 30/HPF, and score 3, > 30/HPF, as described in autoimmune pancreatitis^30^. IgG1-positive cells were scored in the same way.

### Statistical analysis

Continuous data are presented as median and range, and categorical data are presented as a numerical value in each group. While the Mann–Whitney *U* test or unpaired t-test was used to compare continuous variables between the two groups, the χ^2^ test or Fisher’s exact test was used to compare categorical variables. The OS, median, and 95% CIs were determined using the Kaplan–Meier method. The differences between groups were compared using the log-rank test. The univariate Cox proportional hazards regression analysis followed by a multivariate analysis was used to identify the determinants of a high risk of death. Correlations between the groups were examined using the Spearman’s rank correlation test. Variables with a *p*-value < 0.05 in the univariate analyses were considered for inclusion in the multivariate model. Statistical analyses were performed using StatFlex® Version 7.0 (Artech, Osaka, Japan). Statistical significance was established at a *p*-value < 0.05.

### Ethics declarations

This study was approved by the Ethics Committee of the Shinshu University School of Medicine (Approval Number 4205), Hamamatsu University School of Medicine (Approval Number: 2019-0009), Toranomon Hospital (Approval Number: 1773), Prefectural Chubu Hospital (Approval Number: 2018-120) and Kurume University School of Medicine (Approval Number: 18221). This study was performed in accordance with the Declaration of Helsinki and its subsequent amendments. The need for obtaining patient written informed consent was waived, owing to the retrospective nature of this study (the Ethics Committee of the Shinshu University School of Medicine, Approval Number 4205, Nov 6, 2018).

## Supplementary Information

Supplementary Information.

## Data Availability

The datasets generated during and/or analysed during the current study are available from the corresponding author on reasonable request.

## Abbreviations

Blys: B lymphocyte stimulating factor
DIP: Desquamative interstitial pneumonia
DLco: Diffusing capacity of the lung for carbon monoxide
FVC: Forced vital capacity
HPF: High-power field
HRCT: High-resolution computed tomography
Ig: Immunoglobulin
IgG4-RD: Immunoglobulin G4-related disease
IgG4-RRD: Immunoglobulin G4-related respiratory disease
IIPs: Idiopathic interstitial pneumonias
iNSIP: Idiopathic non-specific interstitial pneumonia
IPAF: Interstitial pneumonia with autoimmune features
IPF: Idiopathic pulmonary fibrosis
MDD: Multidisciplinary discussion
OS: Overall survival
SLB: Surgical lung biopsy
UIP: Usual interstitial pneumonia
