# Supplementary material for: Prognostic implication of IgG4 and IgG1-positive cell infiltration in the lung in patients with idiopathic interstitial pneumonia
Source: Sci Rep. 2022 Jun 3;12:9303. doi: 10.1038/s41598-022-13333-8 (PMC9166741; doi:10.1038/s41598-022-13333-8)
Supplement: Supplementary file 1 — Supplementary Information. [file 41598_2022_13333_MOESM1_ESM.docx]

**Prognostic implication of IgG4 and IgG1-positive cell infiltration in the lung in patients with idiopathic interstitial pneumonia**

Masamichi Komatsu^a^, Hiroshi Yamamoto^a*^, Takeshi Uehara^b^, Yukihiro Kobayashi^b^, Hironao Hozumi^c^, Tomoyuki Fujisawa^c^, Atsushi Miyamoto^d^, Tomoo Kishaba^e^, Fumihito Kunishima^f^, Masaki Okamoto^g^, Hideya Kitamura^h^, Tae Iwasawa^i^, Shoichiro Matsushita^j^, Yasuhiro Terasaki^k^, Shinobu Kunugi^k^, Atsuhito Ushiki^a^, Masanori Yasuo^a^, Takafumi Suda^c^, and Masayuki Hanaoka^a^

^a^First Department of Internal Medicine, Shinshu University School of Medicine, Matsumoto, Japan

^b^Department of Laboratory Medicine, Shinshu University School of Medicine, Matsumoto, Japan

^c^Second Division, Department of Internal Medicine, Hamamatsu University School of Medicine, Hamamatsu, Japan

^d^Department of Respiratory Medicine, Respiratory Center, Toranomon Hospital, Tokyo, Japan

^e^Department of Respiratory Medicine, Okinawa Prefectural Chubu Hospital, Uruma, Japan

^f^Division of Pathology, Okinawa Prefectural Chubu Hospital, Uruma, Japan

^g^Division of Respirology, Neurology, and Rheumatology, Department of Internal Medicine, Kurume University School of Medicine, Kurume, Japan

^h^Division of Respiratory Medicine, Kanagawa Cardiovascular and Respiratory Center, Yokohama, Japan

^i^Department of Radiology, Kanagawa Cardiovascular and Respiratory Center, Yokohama, Japan

^j^Department of Radiology, Yokohama City University Hospital, Yokohama, Japan

^k^Department of Analytic Human Pathology, Nippon Medical School, Tokyo, Japan

***Corresponding author:**

Hiroshi Yamamoto, MD, PhD

First Department of Internal Medicine, Shinshu University School of Medicine, 3-1-1 Asahi, Matsumoto 390-8621, Japan

Telephone +81 263 37 2631

Fax +81 263 36 3722

Email: [yama5252@shinshu-u.ac.jp](mailto:yama5252@shinshu-u.ac.jp)

**Supplementary Figure**

**Figure S1. Correlation between serum IgG4 and IgG4-positive cells in the lung.**


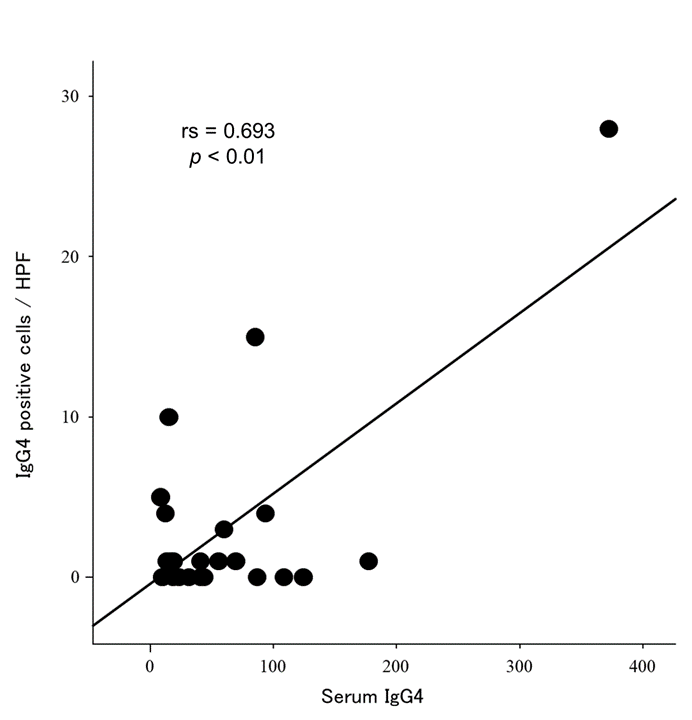


Ig, immunoglobulin; HPF, high-power field.

**Figure S2. Correlation between IgG1-positive cells and IgG4-positive cells in the lung.**


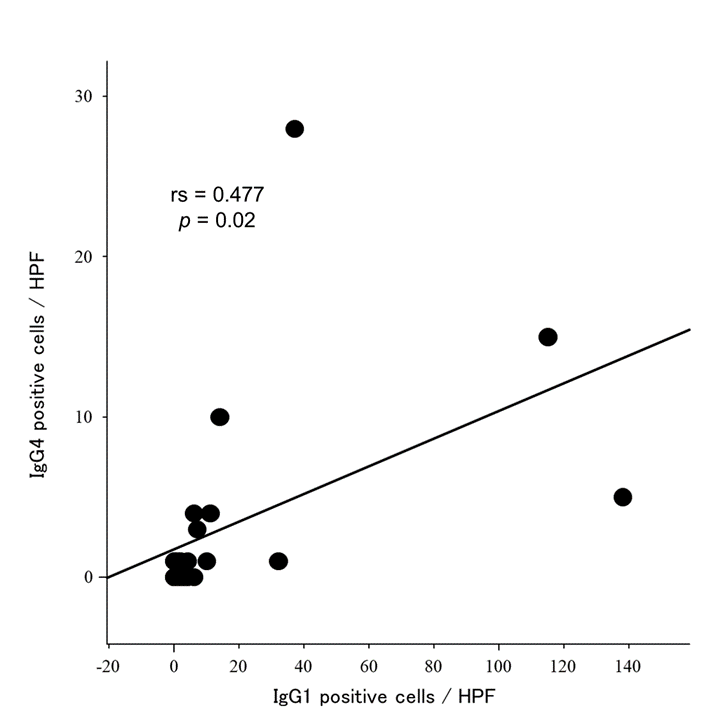


Ig, immunoglobulin; HPF, high-power field.

**Figure S3. Survival curves of patients with idiopathic pulmonary fibrosis (IPF) with or without IgG1-positive cell infiltration (IgG1-positive cells/HPF > 40).**


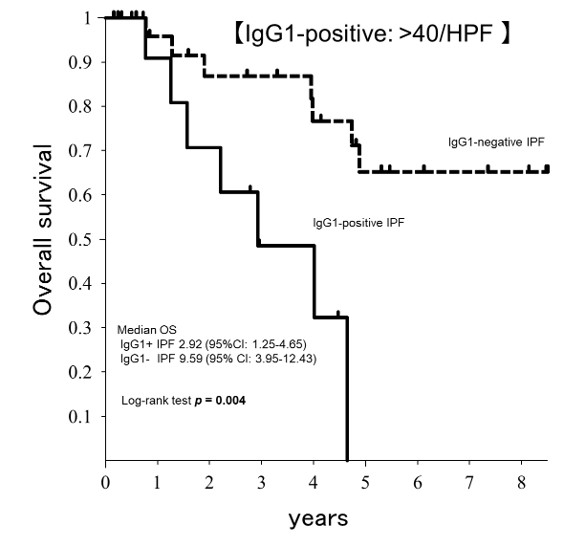


The Kaplan-Meier curve of the overall survival of patients with idiopathic pulmonary fibrosis with or without IgG1-positive cell infiltration in the lungs (IgG1-positive: IgG4-positive cells > 40/HPF). (Solid line: IgG1-positive IPF, Broken line: IgG1-negative IPF).

CI, confidence interval; HPF, high-power field; Ig, immunoglobulin; IPF, idiopathic pulmonary fibrosis; OS, overall survival.

**Figure S4. Histopathological findings.**

**
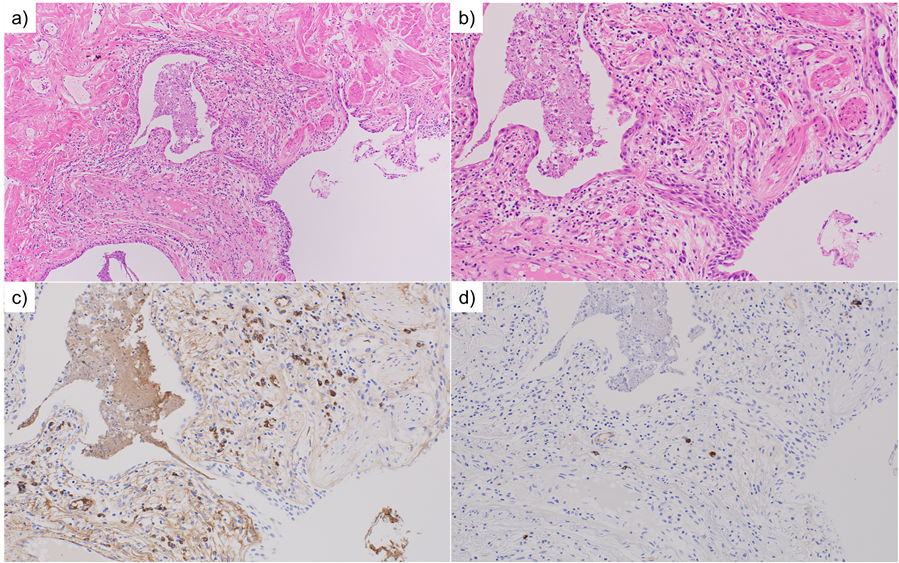
**

**Figure legend.** Representative pathological findings. This patient was diagnosed with idiopathic pulmonary fibrosis with multidisciplinary discussion. (a, b) Inflammatory cell infiltration is seen around the airway (haematoxylin and eosin staining; magnification: 10×, 20×, respectively). (c, d) Images showing an increase in the IgG1-positive cells around the airway, while less frequent in the IgG4-positive cells. (c) IgG1 and (d) IgG4 immunohistochemistry, respectively; magnification: 20×.

**Supplementary Table S1**

**Clinical features and serum autoantibody of patients with idiopathic interstitial pneumonias (IIPs) with or without IgG4-positive cells.**

Data are presented as N. Bold font: *p*-value < 0.05.

ANA, antinuclear antibody; MPO-ANCA, myeloperoxidase-anti-neutrophil cytoplasmic antibody; PR3-ANCA, proteinase-3-antineutrophil cytoplasmic antibody; y/n, yes/no.
